# Necrosis avid near infrared fluorescent cyanines for imaging cell death and their use to monitor therapeutic efficacy in mouse tumor models

**DOI:** 10.18632/oncotarget.5498

**Published:** 2015-10-12

**Authors:** Bangwen Xie, Marieke A. Stammes, Pieter B.A.A. van Driel, Luis J. Cruz, Vicky T. Knol-Blankevoort, Martijn A.M. Löwik, Laura Mezzanotte, Ivo Que, Alan Chan, Jeroen P.H.M. van den Wijngaard, Maria Siebes, Sven Gottschalk, Daniel Razansky, Vasilis Ntziachristos, Stijn Keereweer, Richard W. Horobin, Mathias Hoehn, Eric L. Kaijzel, Ermond R. van Beek, Thomas J.A. Snoeks, Clemens W.G.M. Löwik

**Affiliations:** ^1^ Department of Radiology, Leiden University Medical Center, Leiden, The Netherlands; ^2^ Percuros BV, Enschede, The Netherlands; ^3^*In-vivo*-NMR Laboratory, Max Planck Institute for Neurological Research, Cologne, Germany; ^4^ Department of Biomedical Engineering and Physics, Academic Medical Center, University of Amsterdam, Amsterdam, The Netherlands; ^5^ Faculty of Medicine, Technical University of Munich, Munich, Germany; ^6^ Institute for Biological and Medical Imaging, Helmholtz Center Munich, Munich, Germany; ^7^ School of Life Sciences, College of Medical, Veterinary and Life Sciences, The University of Glasgow, University Avenue, Glasgow, Scotland, UK; ^8^ Medres, Cologne, Germany

**Keywords:** cell death, imaging, cyanines, necrosis avid contrast agents, cancer

## Abstract

Quantification of tumor necrosis in cancer patients is of diagnostic value as the amount of necrosis is correlated with disease prognosis and it could also be used to predict early efficacy of anti-cancer treatments. In the present study, we identified two near infrared fluorescent (NIRF) carboxylated cyanines, HQ5 and IRDye 800CW (800CW), which possess strong necrosis avidity. *In vitro* studies showed that both dyes selectively bind to cytoplasmic proteins of dead cells that have lost membrane integrity. Affinity for cytoplasmic proteins was confirmed using quantitative structure activity relations modeling. *In vivo* results, using NIRF and optoacoustic imaging, confirmed the necrosis avid properties of HQ5 and 800CW in a mouse 4T1 breast cancer tumor model of spontaneous necrosis. Finally, in a mouse EL4 lymphoma tumor model, already 24 h post chemotherapy, a significant increase in 800CW fluorescence intensity was observed in treated compared to untreated tumors. In conclusion, we show, for the first time, that the NIRF carboxylated cyanines HQ5 and 800CW possess strong necrosis avid properties *in vitro* and *in vivo*. When translated to the clinic, these dyes may be used for diagnostic or prognostic purposes and for monitoring *in vivo* tumor response early after the start of treatment.

## INTRODUCTION

Cell death by necrosis merely occurs under pathological conditions, as a result of physiochemical damage or sudden metabolic failure and is involved in cancer development and treatment [1, 2]. The amount of tissue necrosis is of diagnostic value in many cancer types, since a high degree of necrosis is an indicator of rapid and aggressive tumor growth and is often correlated with poor prognosis [3–10]. Moreover, necrosis can also be induced by injury caused to tumor tissue by anti-cancer treatments. Finally, therapeutic approaches that initially induce apoptotic cell death often result in secondary necrosis, as a natural outcome of the complete apoptotic program [11]. Accurate quantification of the amount of tissue necrosis has great potential for pre- clinical and clinical applications, especially in monitoring anti-cancer efficacy at an early stage of treatment instead of at the end of therapy. However, the existing modalities and methods as for example standardized uptake values (SUVs) of FDG-PET, determination of tumor markers or of specific tumor mRNAs all lack the accuracy for a broad and routine application [12, 13]. Therefore, the long lag- time in determining therapy outcome causes loss of valuable treatment time in non-responding patients that will receive expensive treatment and are unnecessarily exposed to side effects. Early evaluation of the therapy efficacy would therefore facilitate the growing call for individualized cancer treatment, allowing the clinician to adjust the therapy based on tumor response, resulting in higher survival rates and cost-efficacy [1, 2].

*In vitro*, cell necrosis is often measured using dyes such as Eosin, Propidium Iodide (PI), TO-PRO-3 and Trypan Blue, which enter necrotic cells upon loss of membrane integrity and cannot permeate living cells. PI and the cyanine TO-PRO-3 subsequently intercalate into DNA [14–16] rendering them potentially mutagenic, which has hampered their clinical use. Perfetto *et al*. showed that amine-reactive cyanines could also be employed to discriminate between living and dead cells *in vitro* [17, 18]. The amine-reactive group on such cyanines can covalently interact with free amino moieties that are available on every protein. Because these amine-reactive compounds are incapable of passing intact cell membranes, only extracellular membrane proteins of living cells are labeled. However, as soon as cells lose their membrane integrity, cytoplasmic proteins become available for dye binding, leading to an accumulation of these agents in dead cells. Though, this principle cannot be employed *in vivo*, as immediately after injection, these reactive cyanines will non-specifically interact with all proteins accessible.

*In vivo*, MRI in combination with non-specific contrast agents, such as Dotarem, have been employed to visualize necrosis. However, with this procedure it was impossible to reliably distinguish healthy from necrotic tissue or neoplastic growth [19]. Consequently, the focus shifted towards the development of compounds that could selectively target necrotic tissues. Already in 1988, Epstein and colleagues developed monoclonal antibodies against nuclear antigens, allowing specific targeting of necrotic tissue present in solid tumors [20]. However, the use of antibodies is limited due to their size, resulting in limited tissue penetration, as well as the induction of unwanted immune responses [21].

Necrosis avid contrast agents (NACAs) are another class of compounds that specifically accumulate in necrotic tissue, these are categorized in porphyrin and non-porphyrin-based compounds. NACAs, such as the well-known compound hypericin, are assumed to specifically bind proteins, peptides and nucleotides that become available upon loss of cell membrane integrity [22, 23]. However, most of these compounds have poor solubility, high tendency to aggregate, are photo-toxic and lack specificity, which are the main reasons why there are currently no clinically approved NACAs available [23, 24]. Therefore, there is an unmet need for non-toxic, small-molecule based probes that can target necrosis with high specificity for diagnostic imaging and treatment follow-up.

In the present study, using several *in vitro*, cell death, assays, we identified the near infrared fluorescent (NIRF) carboxylated cyanines, HQ5 and 800CW as new non-toxic water soluble NACAs. These NACAs bind to intracellular cytoplasmic proteins of cells that have lost membrane integrity. We employed quantitative structure activity relations (QSAR) modelling to predict the overall trajectory of these dyes to their cellular localization sites. In contrast to amine-reactive or maleimide containing cyanines, used for protein labeling, carboxylated cyanines cannot covalently interact and are therefore indicated as non-reactive. Next to *in vitro* studies, we also characterized the necrosis avid properties of HQ5 and 800CW in an *in vivo* 4T1 mouse breast cancer model of spontaneous tumor necrosis and in an EL4 murine lymphoma model in which cell death was induced by chemotherapy. In these animal models, due to their small size, whole body imaging using NIRF imaging, or multi-spectral optoacoustic (OA) imaging, is well suited as a light penetration depth of several cm can be obtained in this part of the spectrum [25]. The actions of the cyanines were compared to those of the blood pool agent 800CW-PEG.

## RESULTS

### QSAR modelling

**Figure 1:**
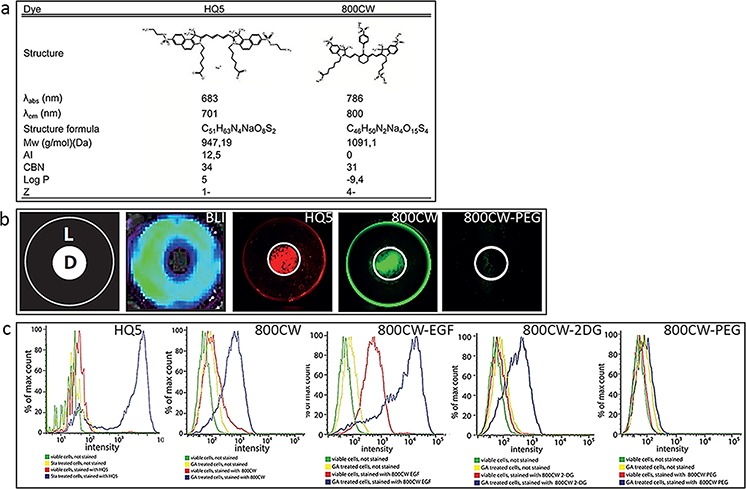
Physicochemical characteristics and *in vitro* examination of the necrotic avid properties of the near infrared fluorophores (NIRF) HQ5 and 800CW **a.** λabs = absorbance wavelength; λem = emission wavelength; Mw = molecular weight; AI = amphiphilic index; CBN = conjugated bond number; logP = log octanol-water partition coefficient; Z = electric charge. **b.** In the dry ice cell death assay an area of necrotic cells was induced in the center of a confluent monolayer of 4T1-luc2 murine breast cancer cells by applying dry ice for 15 sec to the underside of a culture well. Cells in the periphery of the culture well remained alive (schematically represented as, D = dead cells, L = living cells). Cell viability was confirmed by bioluminescent imaging (BLI). After 15 min incubation of the cells with HQ5 or 800CW and subsequent washing, a strong fluorescent signal was obtained from the area of dead cells. The 800CW-PEG signal was almost absent both in the areas of living and dead cells. **c.** FACS analyses performed with viable 4T1-luc2 cells and 4T1-luc2 cells treated with GA (4 uM) or Sta 3 uM) treatment, followed by staining with HQ5 or 800CW. The fluorescence intensity of HQ5 or 800CW stained dead cells was significantly increased compared to that of viable cells. FACS analyses were also performed with viable and GA treated cells, subsequently stained with 800CW-EGF, 800CW-2DG and 800CW-PEG. The fluorescence intensity of both 800CW-EGF and 800CW-2DG, in dead cells, was increased compared to viable cells. Using 800CW-PEG, there was no detectable difference in fluorescence intensity between viable, dead and unstained cells.

Figure 1a shows estimates of the most widely used numerical structure parameters, amphiphilicity index (AI), conjugated bond number (CBN), lipophilicity (logP) and charge (Z), applied to QSAR modelling of the carboxylated cyanines HQ5 and 800CW.

### *In vitro* characterization of necrosis avid cyanines

Using a newly developed *in vitro* cell death assay, based on local killing of cells by freezing [26], we identified the carboxylated cyanines HQ5 and 800CW to exhibit strong necrosis avid and imaging properties. Figure 1b shows a schematic representation of the central, dry ice induced, area of dead 4T1-luc2 cells (D) and the rim of living cells (L) in the periphery. Moreover, bioluminescent imaging (BLI) measurements, indicated that no bioluminescent signals were obtained from the dead cells in the center of the well while the surrounding living cells produced strong signals. In contrast, HQ5 or 800CW incubated wells showed a strong fluorescent signal in the area of dead cells, but not in the area of living cells. The non-specific contrast agent 800CW-PEG, however, showed minimal affinity for dead cells.

The results obtained from the dry ice assay were confirmed by FACS analysis (Figure 1c). 4T1-luc2 cells which were killed by the cytotoxic agents gambogic acid (GA) or Staurosporine (Sta) stained highly positive for HQ5 and 800CW, this in contrast to living cells. Moreover, our FACS experiments showed that the commercially available NIRF imaging probes 800CW-2DG and 800CW-EGF in which 2-Deoxyglucose (2-DG) or epidermal growth factor (EGF) are conjugated to the side chains of 800CW, also specifically accumulated in dead 4T1-luc2 cells However, the non-specific contrast agent 800CW-PEG did not accumulate in dead cells.

**Figure 2:**
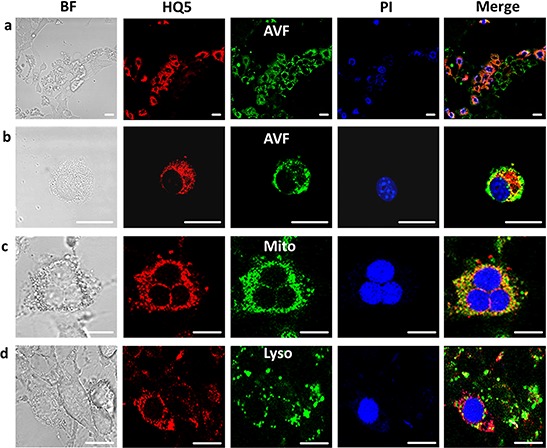
Confocal microscopic images of the localization of HQ5 in gambogic acid treated 4T1-luc2 cells **a.** The bright-field view (BF) image shows the morphology of GA treated cells undergoing cell death. After treatment with GA, cells were stained with HQ5 (red), AVF (green) and PI (blue). A large number of cells stained positive for AVF. The merged image depicts a fluorescence overlay of HQ5, AVF and PI staining, indicating that cells stained with HQ5 were also positive for PI. **b.** At the level of a single necrotic cell it was shown that HQ5, appearing as a granular cytoplasmatic staining, did not co-localize with membrane AVF nor with PI nuclear staining. **c–d.** The granular HQ5 staining was further shown to have a great extent colocalization with Mitotracker (Mito) but not with Lysotracker (Lyso) in GA treated 4T1-luc2 cells. The bar represents 20 μm.

Figure 2a depicts a confocal microscopic image of a GA treated 4T1-luc2 cell culture stained with HQ5, Annexin V-FITC (AVF) and PI. The bright-field (BF) image shows the morphology of GA treated cells undergoing cell death. Most cells stained AVF positive and a few were positive for HQ5 and PI. The HQ5 positively stained cells coincided with PI nuclear staining and not with AVF phosphatidylserine (PS) staining, as visualized in the merged image. The intracellular distribution of HQ5, PI and AVF staining in a single necrotic 4T1-luc2 cell is shown in Figure 2b. PI selectively stained the cell nucleus whereas AVF membrane staining was spread unevenly over the entire cell surface leaving the nucleus unstained. The uneven distribution of the stain may be explained by the loss of membrane integrity. HQ5 showed a more uniform granular staining pattern and did not co-localize with the nuclear stain PI or AVF. Furthermore, Figure 2c–2d shows that the granular HQ5 staining to a great extent co-localizes with Mito-tracker (mitochondria) but not with Lyso-tracker (lysosomes). Moreover, HQ5 also reveals a unique perinuclear staining.

Confocal microscopy could not be performed using 800CW as our system is not suitable for the detection of 800 nm fluorescence.

**Figure 3:**
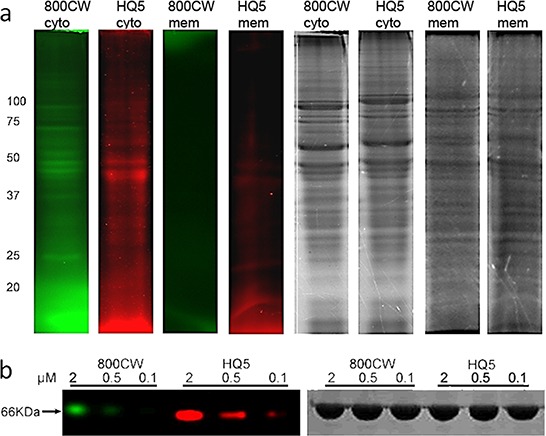
SDS-PAGE analyses of HQ5 and 800CW protein binding **a.** SDS-PAGE gel electropherogram of cytoplasmatic- and membrane fractions of 4T1-luc2 cell lysate, incubated with HQ5 or 800CW. Protein binding of HQ5 and 800CW was observed in the cytoplasmic but not in the membrane fraction. Coomassie blue staining confirmed the presence of proteins in both fractions. HQ5 and 800CW staining showed a different pattern, albeit with some common features. **b.** Binding of HQ5 or 800CW, at different concentrations (0.1, 0.5 and 2 μM), to bovine serum albumin (BSA).

Specific cyanine affinity towards membrane and cytoplasmatic proteins was examined on SDS-PAGE using isolated membrane- and cytoplasmic protein fractions of 4T1-luc2 cells. As shown in Figure 3a, HQ5 and 800CW strongly stained several protein bands in the cytoplasmatic fraction, but not in the membrane fraction. There was some overlap in the staining pattern of the two dyes. Coomassie blue staining indicated that proteins were abundantly present in both fractions.

Furthermore, also affinity of the cyanines towards serum albumin was examined using SDS-PAGE analysis. As depicted in Figure 3b, HQ5 shows a stronger dose-dependent binding to BSA compared to 800CW, although binding only occurred at micromolar concentrations

### Animal model with tumor necrosis

**Figure 4:**
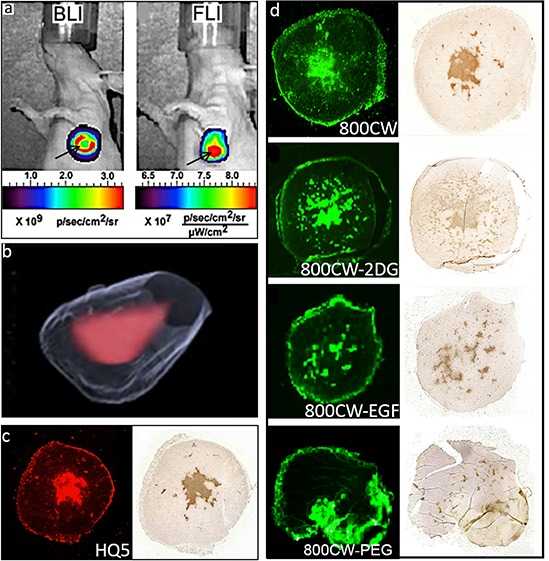
*In vivo* and *ex vivo* imaging of spontaneous 4T1-luc2 tumor necrosis with HQ5, 800CW, 800CW-EGF, 800CW-2DG and, 800CW-PEG **a.** Representative *in vivo* whole body BLI and FLI images of a 4T1-luc2 tumor injected with HQ5. The BLI signal originated from the periphery of the tumor (red ring), whereas the HQ5 FLI signal mainly originated from the necrotic core (red spot). **b.** 3D reconstruction of the localization of HQ5 in a tumor using an automated fluorescence camera mounted on a cryo-microtome. **c.** Images of a HQ5 and corresponding TUNEL stained tumor section. **d.** Images of a 800CW, 800CW-2DG, 800CW-EGF and a 800CW-PEG and their corresponding TUNEL stained tumor sections. In contrast to HQ5, 800CW, 800CW-2DG and 800CW-EGF, 800CW-PEG did not co-localize with TUNEL staining.

The necrosis avid properties of the cyanines were evaluated in an animal model of 4T1-luc2 breast tumors, which, during growth, spontaneously develop a necrotic core. As shown in Figure 4a, the BLI signal obtained from these tumors has a lower intensity in the center compared to the periphery of the tumor, which is indicative of the presence of a necrotic core. As expected, FLI of the cyanine HQ5 showed strong accumulation of fluorescence in the necrotic center of the tumor.

Localization of HQ5 fluorescence in the necrotic core of the tumor was confirmed in 3D reconstructions of cryo-sections (Figure 4b) and showed co-localization with TUNEL staining in parallel paraffin tumor sections (Figure 4c). As shown in Figure 4d, 800CW and bio-conjugated receptor targeting probes 800CW-2DG and 800CW-EGF also strongly accumulated in the necrotic areas of tumors, indicated by a co-localization with TUNEL staining. In contrast, 800CW-PEG did not co-localize with TUNEL staining and most of its fluorescence signal was localized in the tumor periphery.

**Figure 5:**
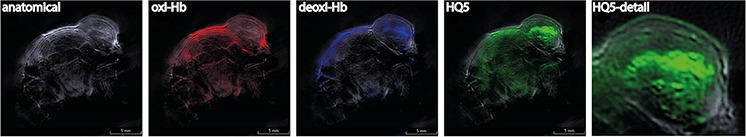
*In vivo* opto-acoustic imaging of necrosis of spontaneous 4T1-luc2 tumor necrosis with HQ5 MSOT measurements showing the single wavelength anatomical image and unmixed signals of oxygenized haemoglobin (oxi-Hb), deoxygenized haemoglobin (deoxi-Hb) and HQ5. Detail of HQ5 targeting of the necrotic core in the center of the tumor.

Furthermore, we employed 3D Multi-Spectral Optoacoustic Tomography (MSOT) imaging to visualize the location of HQ5 in the tumor *in vivo*. As shown in Figure 5, the HQ5 OA signal co-localized with the deoxygenated haemoglobin signal present in the center of the tumor and not with the oxygenated haemoglobin signal present in the viable rim of the tumor, confirming necrotic core localization.

### Monitoring early therapeutic response in tumors

**Figure 6:**
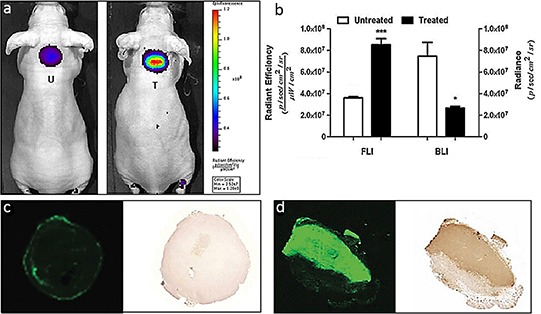
Monitoring anti-tumor efficacy in a EL4-CBG99-luc lymphoma mouse model of chemotherapy **a.** Representative whole body FLI images of tumor bearing mice treated with a combination of CTX and ETO and 24 hr later injected with 800CW. After another 24 h, *in vivo* whole body and *ex vivo* tumor FLI and BLI images were acquired and signal intensities were quantified. **b.** The BLI signals obtained from the treated animals were significantly lower as compared to those of the untreated controls (*p < 0.05). In contrast, the 800CW signals from the treated animals were significantly higher as compared to those of the untreated controls (***p < 0.001). **c–d.** Images of 800CW containing and a TUNEL stained tumor section of an (c) untreated tumor and a (d) treated tumor. The fluorescent signal obtained from a section of a treated tumor co-localized with TUNEL staining of the same section. Negligible 800CW fluorescence and TUNEL staining was observed in the untreated tumor. T = treated; U = Untreated.

We investigated early therapeutic responses in tumors treated with a combination of the chemotherapeutic agents Cyclophosphamide (Cy) and Etoposide (Et) in EL4-CBG99-luc lymphoma bearing mice. For this, control tumor bearing mice and mice treated with Cy/Et were injected with 800CW 24 h after chemotherapy. After another 24 h, fluorescence intensity of the tumors was measured. We observed a 2.4-fold higher fluorescence intensity in the tumors of chemo treated mice compared to those of untreated animals (*p* < 0.001). Vice versa, the mean BLI intensity of untreated tumors was 2.8-fold higher than that of chemo treated tumors (*p* < 0.05) (Figure 6a–6b). Histological examination of the tumors showed a large area of TUNEL positive tissue in the tumors treated (Figure 6c) with chemotherapy which co localized with 800CW staining, whereas, no TUNEL or 800CW positive tissue was present in control tumors (Figure 6d).

## DISCUSSION

Reagents that can monitor necrosis *in vivo* have potential diagnostic and prognostic value in staging of cancer as well as for monitoring early efficacy of anti-cancer therapies [1, 27]. Compared to apoptosis, relatively few studies have addressed the possible role of necrosis as a biomarker for clinical applications. As a result, clinical probes that specifically image or target necrosis are currently unavailable. Consequently, we present two NIRF carboxylated cyanines that display necrosis avidity *in vitro* and *in vivo*. Our dry ice cell death assay showed a selective staining of dead cells using the carboxylated cyanines HQ5 and 800CW. However, no dead cell staining was observed with the macromolecule, 800CW-PEG (25-60 kDa). Nevertheless, the smaller 800CW-conjugated probes, 800CW-EGF (6 kDa) and 800CW-2DG (1 kDa), also selectively stained dead cells. The results obtained in the dry-ice assay were confirmed by FACS analysis. The difference in necrosis avidity between 800CW and its PEG conjugated form might be due to the relatively large size of PEG which may cause steric hindrance, as 800CW is maximally 4% of the mass of the conjugate. In line, de Boer et. al. recently showed that macromolecule cetuximab-IR Dye 800CW was also unable to accumulate in tumor necrosis [28]. The binding to dead cells of the carboxylate forms of 800CW and HQ5 might be unexpected since these compounds, in contrast to the NHS-ester and maleimide forms, do not contain a reactive group and mainly serve as “dye-only” control to examine potential retention of the compound. In addition, for 800CW it has been shown that after i.v. injection it does not retain in the body and is rapidly cleared via the kidneys [29].

Confocal microscopy showed no uptake of any stain in living cells. Nevertheless, after GA treatment we observed HQ5 staining coincided with PI but not with AVF staining. As PI selectively targets cells that have lost membrane integrity, this characteristic is most likely also involved in the dead cell targeting of HQ5. This indicates that HQ5 does not stain apoptotic cells but specifically targets necrotic cells. On a single cell level HQ5 staining did not co-localize with either AVF or PI staining and the granular HQ5 staining pattern appeared to be cytoplasmic. The cellular localization of 800CW could not be examined due to the absence of specific 800 nm microscope settings. We confirmed the cytoplasmatic protein binding of our cyanines by SDS-PAGE analyses using isolated cell membrane and cytoplasmic protein fractions of 4T1-luc2 cells. These fractions were incubated with each dye and we observed that both bound to cytoplasmic and not to the membrane protein fractions. The pattern of protein binding of HQ5 and 800CW to cytoplasmic proteins partly overlapped and also showed preference for a different subset of proteins. This is most likely due to the different physical-chemical properties of these compounds.

Using a QSAR model [30, 31], which is based on the chemical characteristics of HQ5 and 800CW, the expected cellular localization was further explored. The selective accumulation of these dyes in dead cells requires consideration of both dye and cell properties; the former being summarized in Figure 1. The major species of both HQ5 and 800CW under physiological conditions are anionic (Z values of −1 and −4 respectively). Moreover, both dyes have conjugated systems of moderate size (CBN values of 34 and 31). Consequently, the QSAR model predicts both dyes will bind to proteins, albeit not extremely strongly, affinity being due both to ionic attractions with cationic protonated amines and to various non-ionic interactions (van der Waals forces). However, in other respects the dyes differ markedly. Whilst 800CW is extremely hydrophilic (logP = −9.4) and lacks amphiphilicity (AI = 0), HQ5 is lipophilic and extremely amphiphilic (logP = 5.0 and AI = 12.5). Necrotic cells have two characteristic features, which are significant in this context. Their membranes, including those of the plasmalemma and mitochondria, are permeabilized, permitting the passage of impermeable dyes [32, 33]. As a result, the mitochondrial hydrophobic proteins are more easily accessed by dyes entering the necrotic cell. Moreover, protein denaturation will have increased the number of protein molecules with surface hydrophobic domains [34].

The hydrophilic character of 800CW renders it membrane impermeable [31] and so it can only enter permeabilised cells. Once within them, its accumulation will be favored by the enhanced dye-binding commonly found with denatured proteins [35]. As this dye is not amphiphilic, is does not bind significantly to serum albumin. HO5, however, is a lipophilic dye which based on our calculations could possibly enter cells by passive diffusion [31]. The reason that we do not find this in our *in vitro* assay and confocal analysis is most probably due to the fact that HQ5 is very amphiphilic and will bind to serum albumin. It has been shown that amphiphilicity is correlated with serum albumin affinity [31, 36]. Binding to serum albumin of HQ5 was confirmed by us using SDS-PAGE analysis. Once the cells have lost membrane integrity, HQ5 bound to albumin will enter the cells and will bind to proteins with hydrophobic domains, such as those common in mitochondria, or those which are denatured [37]. This is in line with the confocal microscopy data showing that HQ5 for a large part co-localizes with Mitotracker. From the QSAR model it is clear that, although HQ5 and 800CW have different chemical characteristics, both can bind to proteins but probably using different mechanisms. This is also reflected in the difference in binding patterns of the two dyes on SDS-PAGE, showing that they target similar and partly different cytoplasmic proteins. Further detailed studies are needed to elucidate the exact mechanism of binding of the dyes to necrotic cells.

Dyes with large conjugated systems (CBN > 40), but lacking amphiphilicity, such as Coomassie Blue and Evans Blue, are predicted by the QSAR model to bind strongly to all proteins [31]. Such dyes are therefore not expected to show selective uptake into necrotic cells, but will bind to whatever proteins are first contacted.

The question that remains is if, *in vivo*, 800CW and HQ5 specifically target necrotic tissues or that these compounds non-specifically localize in and around necrotic sites because they comprise blood pool characteristics? For example, previous studies indicated that blood pool contrast agents can be employed to indicate tissue injury, due to their passive leakage from blood vessels at sites of tissue damage. In addition, blood pool agents are employed to detect tumors because of their ability to accumulate in tumor tissue, as a result of a process known as the enhanced permeability and retention (EPR) effect [38]. This process is characterized by the ability of macromolecules (>20 kDa), or small molecules bound to serum albumin, to accumulate in tumors as a result of their passive leakage from abnormal tumor vasculature. In contrast, small molecules (<20 kDa), which possess no affinity for blood proteins, do not retain and rapidly penetrate the interstitial space of tumors and subsequently diffuse freely back into the blood pool or the lymphatic system. Therefore, the increased retention of blood pool agents at or in the vicinity of tissue damage sites or in tumors is not due to a specific interaction with necrotic tissue, but is merely the result of reduced diffusion velocity of large molecules out of the tissue compartment.

Previous studies and our study show that 800CW possesses very low affinity for blood proteins and consequently rapidly extravasates after i.v. injection [39]. This indicates that the small molecule 800CW is not a blood pool agent and, therefore, will retain in tissue because of a specific binding to intracellular proteins of necrotic cells. The *in vivo* specificity of 800CW for necrotic tissues is strengthened by the observation that this compound strongly co-localizes with TUNEL staining, a feature that is not observed with the blood pool agent 800CW-PEG. Similar to 800CW, HQ5 also shows a strong co-localization with TUNEL staining in necrotic areas in tumors. However, in contrast to 800CW, HQ5 can bind to serum albumin and thus potentially serves as blood pool agent. Similarly, the photosensitizer Hypericin, which is currently under pre-clinical investigation because of its necrosis avid properties, also possesses affinity for albumin [40]. In contrast, the well-known blood pool agent Evans Blue, which also strongly binds to serum albumin, has been shown to target the viable rim of tumors rather than the necrotic core [41]. Moreover, based on the observation that of the albumin binding compounds Gadophrin-2 and MP2269, only the first possessed NACA properties Ni *et al*. [23] stated that necrosis-avidity is an outstanding feature beyond the general pharmacological process of albumin-binding mediated drug transportation. Combined, it might be concluded that the role of blood protein binding in the mode of action of this particular group of NACAs needs, to be established.

In our 4T1-luc2 tumor necrosis model, HQ5 and 800CW showed co-localization with TUNEL staining, and the same was true for the 800CW-EGF and 800CW-2DG conjugated probes and not for 800CW-PEG. Therefore, it is important to note that 800CW-EGF and 800CW-2DG, that are specifically designed and have been extensively used to target the EGF receptor (EGFR) and the Glucose receptor-1 (GLUT) [42, 43], also have strong necrosis avidity due to the presence of CW800 which can direct these probes towards necrotic cells. This new finding has to be taken into consideration when interpreting experimental results obtained with 800CW-EGF and 800CW-2DG [29, 44–46].

The necrosis avidity of HQ5 and 800CW was further investigated in a well-known model of chemotherapy. We showed that 800CW could be used to monitor early treatment efficacy. This feature is of great significance since in current clinical practice the efficacy of anti-cancer treatment can only reliable be established late during treatment or after completion of the treatment. Therefore, currently non-responding patients receive an expensive treatment and are unnecessarily exposed to side effects [1, 2]. Finally, it is worth mentioning some of the limitations of the present study, which include the use of athymic mice, the use of transplanted and not spontaneous tumors and the usage of only one type of anti-cancer treatment, namely chemotherapy.

### Future perspectives and clinical relevance

OA imaging, a technique in which a pulsating light signal is transformed into an ultrasound wave, provides much deeper tissue penetration (approximately 5 cm) and higher resolution than other optical imaging modalities currently available. This technology can revolutionize medical imaging in clinical practice. With the development of a handheld MSOT scanner, with applications in breast and melanoma imaging, the clinical translation of OA imaging is already materialized and may also ease the translation of our necrosis probes to the clinic especially since we have shown that HQ5 can be detected using MSOT [47–50].

However, for detection of tumors deep within the body, when measurements beyond the maximal optical or OA penetration depth are required, the dyes have to be radiolabeled in order to allow their visualization with standard clinical imaging modalities like SPECT or PET. Our preliminary results show that radiolabeling of a structural analogue of HQ5 with Indium-111, using the chelate DTPA is feasible and that this probe still specifically accumulates in necrotic cells *in vitro* and in necrotic cores of tumors (unpublished data). Moreover, from an economical point of view it is worth to mention that the production costs of the cyanines, especially when synthesized in bulk amounts are low and that the rates for radio labeling and subsequent SPECT/PET scanning will be comparable to those of other clinically used SPECT/PET probes.

The concept of employing tissue necrosis, as a biomarker for diagnostic and prognostic purposes of disease, is not new. With the objective to target and image necrotic tissue, already back in 1988 Epstein and colleagues developed several so called TNT antibodies [20] and more recently Ni and colleagues [22, 40, 51, 52] reported on the specific necrosis avid properties of the photosensitizer Hypericin in small animals. However, both compounds were examined not just for their potential as contrast agents, but also for their possible usage in cancer treatment by coupling of Iodine-131 used for local radionuclide therapy. In this way, when the necrosis avid agent has accumulated in the necrotic core of the tumor, the cancer is selectively irradiated and killed from the inside. After showing proof of concept, in animal studies [53], the TNT antibodies even reached clinical phase I and II studies [54]. However, for both TNT antibodies and Hypericin, full clinical translation is hampered because of increased concern about adverse effects and other drawbacks based on their physical-chemical properties [54, 55]. No such concerns are expected with the employment of the NIRF cyanines examined in this study. NIRF cyanines are successfully used already for more than a decennium for experimental and clinical experimental purposes including fluorescence image guided surgery without serious side effects [56]. In addition, toxicity studies by Marshall and colleagues, showed that 800CW carboxylate administrated as a single intravenous or intradermal dose of 20 mg/kg, which is about 100-fold above the maximum dose utilized in our experiments, did not result in any pathological evidence of toxicity in rats [29]. Therefore, our necrosis avid carboxylated NIRF dyes can potentially be used clinically to image necrotic tissue for diagnostic and prognostic purposes, to detect treatment response in tumors and for drug delivery.

In conclusion, we have demonstrated that the carboxylated cyanines 800CW and HQ5, as well as 800CW-2DG and 800CW-EGF, possess strong necrosis avid properties. The molecular mechanism of necrosis avidity involves targeting of cytoplasmic proteins after loss of cell membrane integrity. Using NIRF imaging in different mouse models of cancer, we showed that these dyes can be applied to detect spontaneous tumor necrosis, which is of diagnostic and prognostic value. Moreover, we showed that they can be utilized to monitor early treatment responses in tumors after anti-cancer therapy and potentially they can also be used for drug delivery. Therefore, when translated to the clinic, these compounds might become of great value in cancer diagnostics and treatment.

## MATERIALS AND METHODS

### QSAR modelling

Estimation of the structure parameters for carboxylated HQ5 and IRDye 800CW (800CW) and the integration of the parameters with appropriate QSAR models was carried out as detailed elsewhere [30, 31].

### Cyanines

HQ5 carboxylate was obtained from Ilumicare BV (Rotterdam, The Netherlands). The dyes 800CW, 800CW-2-Deoxyglucose (800CW-2DG), 800CW-epidermal growth factor (800CW-EGF) and 800CW-polyethylene glycol (800CW-PEG) were obtained from LI-COR Biosciences.

### Cells and culture conditions

4T1-luc2 murine mammary cancer cells (PerkinElmer) were cultured in complete RPMI-1640 medium (Life Technologies, Inc.). EL4 murine lymphoma cells were cultured in complete Iscove's Modified Dulbecco's Medium (Life Technologies). Cells were transduced with a lentivirus for the expression of CBG99 luciferase under the control of the constitutive promoter PGK as described previously to create EL4-CBG99-luc [57].

### Dry ice dead cell assay

*In vitro*, cell death was studied using a cryo-induced cell death assay, which detailed procedures have been described previously [26]. Briefly, a bar of dry ice was applied to the underside of the culture well confluent with 4T1-luc2 cells for 15 sec. Subsequently, the cells were incubated with HQ5, 800CW or 800CW-PEG, respectively (100 nM, 15 min, room temperature (RT), in the dark). After gentle washing with PBS, the samples were scanned for fluorescence imaging (FLI) using an Odyssey Infrared Imager 9120 (LI-COR). For bioluminescence imaging (BLI), D-luciferin solution (25 μg/μl; SynChem Inc.) was added for 10 min incubation. BLI measurements were then acquired using an IVIS Spectrum imaging system (PerkinElmer).

### FACS analyses

The detailed procedures for flow cytometry of cells after inducing cell death have been described previously [26]. Briefly, 4T1-luc2 cells were incubated in the presence or absence of gambogic acid (GA, 4 μM, 24 h, Calbiochem) or Staurosporine (Sta, 3 μM, 24 h, Sigma-Aldrich). Cells were then collected and re-suspended in 100 μl PBS. The cell suspensions were incubated in the dark for 15 min at RT with one of the cyanines (200 nM). Alternatively, cells were stained with the commercially available cell death probes AVF and PI (PromoKine) in accordance with the manufacturer's protocols. Flow cytometry was performed using a BD LSR II or Canto II Flow Cytometer (BD Biosciences). The data was analyzed using FlowJo software.

### Confocal microscopy

4T1-luc2 cells were cultured in a glass bottomed culture dish (MatTek Corp.) until 80% confluent. Cell death was induced by incubation with GA (3 μM, 1 h). Subsequently, the cells were washed gently with PBS and incubated in the presence of 80 nM HQ5 in the dark for 15 min at RT. AVF and PI were used in accordance with the manufacturer's protocols. Afterwards, samples were imaged using a Leica TCS SP5 confocal microscope (Leica).

### SDS-PAGE analyses

Cytoplasmic and membrane protein samples of 4T1-luc2 cells were prepared using a subcellular protein fractionation kit (Thermo Scientific) according to the manufacturer's protocol. Samples of protein extracts (2 μg per lane) were incubated with either HQ5 or 800CW (1 μM) for 15 min at RT in the dark. For Bovine serum albumin (BSA) commercial preparations of BSA (5 μg per lane, Life Technologies) were incubate with either HQ5 or 800CW (0.1, 0.5 and 2 μM, respectively) for 15 min at RT in the dark. Subsequently, samples were mixed with SDS-PAGE sample buffer without indicative blue dye and loaded onto reduced 12.5% SDS polyacrylamide gel. The precision plus protein marker (BIO-RAD) was loaded to one extra lane. After running SDS-PAGE, the gel was processed for FLI using the Odyssey Infrared Imager 9120 scanner. Finally, protein samples were stained with Coomassie brilliant blue staining (BIO-RAD) and photographed.

### Animals

Female athymic mice (BALB/c *nu*/*nu*, 6 weeks old) were acquired from Charles River Laboratories (L'Arbresle Cedex, France). All experimental procedures were performed under isoflurane gas anesthesia (3% induction, 1.5–2% maintenance) in 70% pressurized air and 30% O_2_, unless stated differently. Animals were sacrificed by cervical dislocation at the end of the experimental period. The animals were housed per 4–5 animals in individually ventilated cages with *ad libitum* access to food and water. All animal experiments were assessed for animal health & ethics and approved by the Animal Welfare Committee of Leiden University Medical Center, the Netherlands. All mice received humane care and were kept in compliance with the *Code of Practice Use of Laboratory Animals in Cancer Research* (Inspectie W&V, July 1999).

### Spontaneous tumor necrosis model

Mice (*n* = 5) received orthotopic inoculations of 2 × 10^4^ 4T1-luc2 cells beneath the upper mammary fat pad. Trypan Blue (Sigma-Aldrich) exclusion was used to examine the viability of the tumor cells before injection. After three weeks, tumors were formed, containing a spontaneous necrotic core. Whole body BLI and FLI measurements were performed using the IVIS Spectrum, with either 10 min post D-luciferin (150 mg/kg) per intraperitoneal (i.p.) injection or 24 h post HQ5 (2 nmole per mouse) per i.v. injection.

3D fluorescent cryomicrotome imaging of a tumor was reconstructed according the previous published methods [58]. In brief, tumor samples were immersed in carboxymethylcellulose sodium solvent (Brunschwig Chemie, Amsterdam, The Netherlands) mixed with 5% Indian ink (Royal Talens, Apeldoorn, The Netherlands) and frozen for at least 24 hrs at −25°C. After each cut, tumor epi-illumination outline images were acquired at an excitation wavelength of 440 nm/20 nm (central wavelength and bandwidth) and an emission wavelength of 435 nm/25 nm, with 300 ms illumination time. Images of tumor fluorescence were acquired at an excitation wavelength of 640 nm/50 nm and an emission wavelength of 712 nm/75 nm, 5000 ms illumination. All tissue samples were imaged in one session with camera binning set at 2048 × 2048 pixel resolution, with a corresponding in-plane resolution of 17 μm. Prior to further analysis, images were converted to 8 bit grey scale.

### MSOT imaging

A group (*n* = 3) of female athymic nude-Fox1nu mice was inoculated with 4T1 cells and subjected to MSOT measurements. MSOT measurements were performed using the inVision 256-TF system (iThera Medical GmbH, Munich, Germany) according to the protocol described previously [59, 60]. In brief, the mice were anesthetized and placed in supine position in the animal holder throughout the entire imaging process. Cross-sectional multispectral OA image datasets were acquired through the tumor at eight different wavelengths in the NIR window (690, 700, 710, 740, 760, 780, 800 and 900 nm). MSOT datasets are reconstructed using the interpolated model-matrix inversion. Afterwards, linear spectral unmixing is applied to each set of multiwavelength images to resolve biodistribution of the different tissue chromophores and the contrast agent, i.e. oxygenized and deoxygenized haemoglobin and HQ5 [61].

### Chemotherapy of murine lymphomas

Two randomized groups mice (*n* = 3) received a subcutaneous (s.c.) inoculation with 1 × 10^5^ EL4-CBG99-luc murine lymphoma cells on the upper back. 11 days after inoculation, the animals either received chemotherapy consisting of i.p. injection of a combination of cyclophosphamide (CTX, 100 mg/kg; Baxter BV, The Netherlands) and etoposide (ETO, 70 mg/kg; Pharmachemie BV, The Netherlands) or remained untreated. After 24 h, all animals received an i.v. injection of 800CW (5 nmole per mouse). FLI measurements were performed another 24 h after injection using the IVIS spectrum. Whole body BLI measurements were performed, before and 24 h after injection of the chemotherapeutic agents, 10 min after i.p. administering D-luciferin (150 mg/kg). After FLI, all mice were sacrificed and the tumors were surgically excised for *ex vivo* FLI and processed for histological analysis. Image analyses were performed using the Living Image software. For quantitative analysis, regions of interest (ROI) from acquired images were selected to cover the tumor regions. Statistical analysis of the average fluorescent radiant efficiency in ROIs was performed using a Student's *t*-test.

### Histopathology analysis

4T1-luc2 and EL4-CBG99-luc tumors were fixed in 4% formaldehyde and embedded in paraffin. 5 μm sections were prepared and imaged for FLI using the Odyssey Infrared Imager 9120 scanner. Afterwards, the consecutive sections were subjected to TdT-mediated dUTP Nick-End Labelling (TUNEL) staining (Promega) to validate accumulation of the NIRF probes in dying and dead cells.
